# Grafted c-kit^+^/SSEA1^−^ eye-wall progenitor cells delay retinal degeneration in mice by regulating neural plasticity and forming new graft-to-host synapses

**DOI:** 10.1186/s13287-016-0451-8

**Published:** 2016-12-30

**Authors:** Xi Chen, Zehua Chen, Zhengya Li, Chen Zhao, Yuxiao Zeng, Ting Zou, Caiyun Fu, Xiaoli Liu, Haiwei Xu, Zheng Qin Yin

**Affiliations:** 1 Southwest Hospital/Southwest Eye Hospital, Third Military Medical University, Chongqing, 400038 China; 2 Key Lab of Visual Damage and Regeneration & Restoration of Chongqing, Chongqing, 400038 China; 3 School of Medicine, Nankai University, Tianjin, 300071 China; 4 Division of Pulmonary and Critical Care Medicine, Department of Medicine, Brigham and Women’s Hospital and Harvard Medical School, Boston, MA 02115 USA; 5 Department of Pediatric Newborn Medicine, Brigham and Women’s Hospital and Harvard Medical School, Boston, MA 02115 USA

**Keywords:** Retinal degeneration, c-kit, Differentiation, Transplantation, Synapse formation, Neuroplasticity

## Abstract

**Background:**

Despite diverse pathogenesis, the common pathological change observed in age-related macular degeneration and in most hereditary retinal degeneration (RD) diseases is photoreceptor loss. Photoreceptor replacement by cell transplantation may be a feasible treatment for RD. The major obstacles to clinical translation of stem cell-based cell therapy in RD remain the difficulty of obtaining sufficient quantities of appropriate and safe donor cells and the poor integration of grafted stem cell-derived photoreceptors into the remaining retinal circuitry.

**Methods:**

Eye-wall c-kit^+^/stage-specific embryonic antigen 1 (SSEA1)^−^ cells were isolated via fluorescence-activated cell sorting, and their self-renewal and differentiation potential were detected by immunochemistry and flow cytometry in vitro. After labeling with quantum nanocrystal dots and transplantation into the subretinal space of rd1 RD mice, differentiation and synapse formation by daughter cells of the eye-wall c-kit^+^/SSEA1^−^ cells were evaluated by immunochemistry and western blotting. Morphological changes of the inner retina of rd1 mice after cell transplantation were demonstrated by immunochemistry. Retinal function of rd1 mice that received cell grafts was tested via flash electroretinograms and the light/dark transition test.

**Results:**

Eye-wall c-kit^+^/SSEA1^−^ cells were self-renewing and clonogenic, and they retained their proliferative potential through more than 20 passages. Additionally, eye-wall c-kit^+^/SSEA1^−^ cells were capable of differentiating into multiple retinal cell types including photoreceptors, bipolar cells, horizontal cells, amacrine cells, Müller cells, and retinal pigment epithelium cells and of transdifferentiating into smooth muscle cells and endothelial cells in vitro. The levels of synaptophysin and postsynaptic density-95 in the retinas of eye-wall c-kit^+^/SSEA1^−^ cell-transplanted rd1 mice were significantly increased at 4 weeks post transplantation. The c-kit^+^/SSEA1^−^ cells were capable of differentiating into functional photoreceptors that formed new synaptic connections with recipient retinas in rd1 mice. Transplantation also partially corrected the abnormalities of inner retina of rd1 mice. At 4 and 8 weeks post transplantation, the rd1 mice that received c-kit^+^/SSEA1^−^ cells showed significant increases in a-wave and b-wave amplitude and the percentage of time spent in the dark area.

**Conclusions:**

Grafted c-kit^+^/SSEA1^−^ cells restored the retinal function of rd1 mice via regulating neural plasticity and forming new graft-to-host synapses.

**Electronic supplementary material:**

The online version of this article (doi:10.1186/s13287-016-0451-8) contains supplementary material, which is available to authorized users.

## Background

As an extension of the central nervous system (CNS), the mammalian neural retina consists of neurons and glial cells. It lacks significant regenerative capacity after development is completed. Consequently, degeneration and loss of photoreceptors or their supporting cells usually results in permanent visual impairment. Of all cases of blindness in the developed world, direct or indirect injury to photoreceptors accounts for approximately 50% [1–3]. Inherited diseases, including retinitis pigmentosa (RP) and Stargardt disease, can produce direct photoreceptor loss. Age-related macular degeneration (AMD), which usually affects aged adults, leads to photoreceptor loss secondary to the death of the retinal pigment epithelium (RPE) and the loss of its supportive role [4]. Although these diseases have diverse causes, the common outcome is photoreceptor loss. However, the underlying part of the retina may still remain largely intact [5, 6]. It has been reported that 80% of bipolar cells still remained in the macular area even at very late stages of RP [7], which makes it possible to restore vision by replacing nonfunctional photoreceptors.

Therapeutic strategies for retinal repair include neuroprotection, anti-inflammatory agents, gene correction, and cell-based therapy [8]. Cell-based therapy encompasses both delivering stem/progenitor cells or their progeny into the degenerating retina and inducing endogenous cellular regeneration, reactivating dormant repair mechanisms to generate new photoreceptors [9–11]. Stem cell-based treatment for retinal degeneration usually functions via the following mechanisms: cell replacement, trophic support, immunomodulation, and synaptic reestablishment [12–15]. As a promising approach for late-stage photoreceptor rescue, cell-based strategies do not interfere with the progression of the disease, instead generating new neurons that integrate into the retinal circuitry to rebuild synaptic connections, which is crucial for long-term efficacy [16–18]. To date, several reports have shown that newborn photoreceptors from post-mitotic photoreceptor precursors can morphologically integrate into the existing circuitry [19–21].

A good cell surface marker or combination of cell markers is usually crucial for isolating stem cells from tissues, with the goals of maintaining a pure population and removing the early-stage cells that pose a risk of tumor formation. c-kit^+^ cells have been shown to be self-renewing, clonogenic, and multipotent both in vitro and in vivo in hearts, lungs, and other organs [22–24]. Furthermore, c-kit and its ligand, stem cell factor, are both expressed in the CNS and the peripheral nervous system [25–28], as well as in the retinas of humans and mice [29–32]. Purified cells expressing c-kit as a surface marker might have potential future applications for the treatment of retinal degeneration diseases.

Preliminary evidence has indicated that c-kit^+^ cells isolated from humans have potential therapeutic value [29]. However, due to the limitations on combining human cells and a retinal degeneration rat model, the formation of synapses between the grafted cells and recipient retinal cells could not be determined. Thus, in our present study, we evaluated whether administration of c-kit^+^ cells could rescue the visual function of mice with retinal degeneration and, more importantly, whether the transplanted cells could integrate into the host retina and form synapses.

## Methods

### Mice

*C57BL/6 J* and *B6.C3-Pde6b* ^*rd1*^ *Hps4* ^*le*^ (rd1) mice were maintained in the animal facility of Third Military Medical University, Chongqing, China. All experiments were conducted according to the guidelines for laboratory animal care and use of Third Military Medical University. The mice were kept on a standard 12-hour/12-hour light–dark cycle. All of the related experiment procedures met the requirements of Laboratory Animal Welfare and Ethics Committee of Third Military Medical University.

### Isolation and culture of mouse eye-wall progenitor cells

Briefly, the mice were sacrificed on postnatal day (PND) 1, and the eyes were dissected out and rinsed in phosphate-buffered saline (PBS; Corning Inc., Corning, NY, USA). The cornea, lens, vitreous body, and connective tissue attached to the eye shell were removed. The eye shells were chopped into small pieces and incubated in PBS containing collagenase I (10 mg/ml; Worthington Biochemical, Lakewood, NJ, USA) and collagenase II (25 mg/ml; Worthington Biochemical). The dissociated cells were filtered through a 40-μm filter (BD Biosciences, Franklin Lakes, NJ, USA) and seeded in growth medium containing DMEM/F12 medium (Lonza Biologics, Hopkinton, MA, USA) supplemented with fetal bovine serum (FBS, 10%; Thermo Fisher Scientific, Waltham, MA, USA), murine basic fibroblast growth factor (bFGF, 20 ng/ml; PeproTech, Rocky Hill, NJ, USA), murine epidermal growth factor (EGF, 20 ng/ml; PeproTech), insulin/transferrin/sodium selenite (1:500; Lonza Biologics), and leukemia inhibitor factor (10 ng/ml; EMD Millipore, Billerica, MA, USA).

All of the PND 1 pups from one pregnant mother (usually about 4–7 pups) were harvested for single cell isolation. The cell isolation experiment was repeated five times. These primary isolated cells were plated on the Petri dishes and were sorted for c-kit^+^/stage-specific embryonic antigen 1 (SSEA1)^−^ population by fluorescence-activated cell sorting (FACS) when the cells reached confluence (only one passage).

### FACS of the eye-wall c-kit^+^/SSEA1^−^ progenitor cells

For c-kit^+^/SSEA1^−^ cell isolation, cells were detached using HyQTase (Thermo Fisher Scientific), blocked with Fc (BioLegend, San Diego, CA, USA) for 15 min, and then incubated with anti-mouse c-kit antibody conjugated with APC (BioLegend) and anti-mouse SSEA1 antibody conjugated with FITC (BD Biosciences) at 4 °C for 30 min. After rinsing with staining buffer (eBioscience, San Diego, CA, USA), the cells were purified for the c-kit-positive, SSEA1-negative population using a FACSAria Flow Cytometer (BD Bioscience). The purified cells were passaged five times before differentiation assays and cell transplantation.

### Limiting dilution and clone formation

The limiting dilution protocol was based on our previous work [33]. Briefly, 100 mouse eye-wall c-kit^+^/SSEA1^−^ cells were plated in a 100 mm diameter dish (a density of ≈ 1 cell/60 mm^2^). The clones were formed at approximately 2–3 weeks after plating.

### Growth analysis

In brief, 10,000 cells were plated and counted daily for 7 days. On the 7th day, 5-bromo-2′-deoxyuridine (BrdU) labeling was assessed by applying BrdU Labeling and Detection Kit I (Roche, Penzberg, Upper Bavaria, Germany). According to the manufacturer’s instructions, BrdU was added to the growth medium (final concentration 10 μM) and the cells were incubated for 1 hour. After the cells were fixed, cells were incubated with anti-BrdU antibody (1:10) at 37 °C for 1 hour, and then incubated with fluorophore-conjugated secondary antibodies (1:10) for 1 hour at 37 °C. Nuclei were counterstained with 4′,6-diamidino-2-phenylindole (DAPI). At same time point, the apoptosis of c-kit^+^/SSEA1^−^ cells was analyzed in vitro by terminal deoxy nucleotidyl transferase-mediated nick end labeling (TUNEL) assay using an In Situ Cell Death Detection Kit (Roche). According to the manufacturer’s instructions, cells were fixed, permeabilized, and incubated with the mixture of enzyme solution (TdT) and Label Solution (fluorescein-dUTP; 1:9) for 1 hour at 37 °C. The nuclei of the cells were counterstained with DAPI.

### Differentiation characterization assay

Cell differentiation protocols were modifications of methods described previously [34]. To induce cell differentiation, c-kit^+^/SSEA^1^− cells were cultured in differentiation medium, which contained DMEM/F12 (Lonza Biologics) supplemented with bFGF (10 ng/ml; PeproTech) and B27 (1:50; Thermo Fisher Scientific), for the first 2 days. For amacrine cell differentiation specifically, cells were switched to differentiation medium plus JAG1 (40 nM; AnaSpec, Fremont, CA, USA) for another 6 days. For horizontal cell differentiation, cells were cultured in differentiation medium plus nerve growth factor (NGF, 10 ng/ml; Sigma-Aldrich, Natick, MA, USA) and insulin-like growth factor 1 (IGF-1, 10 ng/ml; Sigma-Aldrich) for another 6 days. For photoreceptor differentiation, cells were cultured in differentiation medium plus N2 (1:100; Thermo Fisher Scientific), docosahexaenoic acid (DHA, 50 nM; Sigma-Aldrich), retinoic acid (2 μM; Sigma-Aldrich), and γ-secretase inhibitor (DAPT, 10 μM; Sigma-Aldrich) for 2 days and then in medium consisting of DMEM/F12 with B27 (1:50), NGF (10 ng/ml), IGF-1 (10 ng/ml), and brain-derived neurotrophic factor (BDNF, 10 ng/ml; Sigma-Aldrich) for another 4–6 days. For all other cell types, cells were switched to the differentiation medium plus N2 (1:100) for another 6 days.

For RPE cell differentiation, cells were cultured in DMEM/F12 (Lonza Biologics) with 20% knockout serum replacement (Thermo Fisher Scientific), 2 mM glutamine (Thermo Fisher Scientific), and MEM nonessential amino acids solution (1:100; Thermo Fisher Scientific). The medium was changed every 2–3 days.

For smooth muscle cell differentiation, cells were cultured in Medium 231 (Thermo Fisher Scientific) with Smooth Muscle Differentiation Supplement (1:100; Thermo Fisher Scientific) and FBS (5%) for 7 days. For endothelial cell differentiation, cells were cultured in Endothelial Cell Growth Medium-2 Basal Medium (Lonza Biologics) for 7 days.

### Flow cytometry

Flow cytometry was used to identify c-kit^+^/SSEA1^−^ cells and differentiated cells and was performed as described previously [29, 33, 35]. Briefly, cells cultured in growth medium or differentiation medium were detached using HyQTase (Thermo Fisher Scientific) and collected. For surface markers, cells were blocked with CD32/16 (BioLegend) and then incubated with primary antibodies (1:30) or isotype control (1:30; BioLegend) for 30 min at 4 °C. After each procedure, cells were rinsed with staining buffer (eBioscience). For intracellular and nuclear markers, cells were fixed with fixation buffer (eBioscience), blocked with 1% serum, and incubated with primary antibodies (1:30) at 4 °C for 30 min and then with fluorophore-conjugated secondary antibodies (1:30) at 4 °C for 30 min. After each procedure, cells were rinsed with permeabilization buffer (eBioscience). Cells were counted using a FACSCalibur Flow Cytometer and at least 10,000 events were collected for each sample and analyzed using FlowJo software (FlowJo, Ashland, OR, USA).

### Immunocytochemistry

Immunohistochemistry was performed as described previously [33, 35, 36]. Briefly, mouse eyeballs were prefixed in prefixation buffer (5% acetic acid, 0.4% paraformaldehyde, 0.315% saline, and 37.5% ethanol), followed by fixation in 4% paraformaldehyde at 4 °C overnight, and then embedded in paraffin. Sections (5 μm) were stained for further analysis. After being deparaffinized, rehydrated, and boiled in 10 mM citrate buffer, sections were incubated with 10% goat serum and then primary antibodies at 4 °C overnight followed by species-matched fluorophore-conjugated secondary antibodies for 1 hour at 37 °C. Nuclei were stained with DAPI.

For cytoimmunofluorescence staining, cells were fixed with 4% paraformaldehyde, incubated with 5% goat serum and 0.1% Triton X-100, followed by primary antibodies at 4 °C overnight, and then incubated with fluorophore-conjugated secondary antibodies for 1 hour at 37 °C. Nuclei were stained with DAPI. Cells and sections were analyzed using a confocal microscopy system (Leica Camera, Wetzlar, Germany).

The primary antibodies used were as follows: anti-c-kit at 1:200 (Cell Signaling Technology, Danvers, MA, USA), anti-nestin at 1:200 (Abcam, Cambridge, MA, USA), anti-retina homeobox protein Rx (Rax) at 1:200 (Abcam), anti-SRY (sex determining region Y)-box 2 (Sox2) at 1:500 (Abcam), anti-orthodenticle homeobox 2 (Otx2) at 1:400 (Abcam), anti-paired box protein 6 (Pax6) at 1:200 (Abcam), anti-Ki67 at 1:250 (Abcam), anti-telomerase reverse transcriptase (TERT) at 1:200 (EMD Millipore), anti-recoverin at 1:1000 (EMD Millipore), anti-rhodopsin at 1:1000 (Abcam), anti-protein kinase C alpha (PKCα) at 1:250 (Abcam), anti-calbindin at 1:200 (Abcam), anti-glutamate decarboxylase 65 & 67 (GAD) at 1:500 (Abcam), anti-choline acetyltransferase (ChAT) at 1:100 (Abcam), anti-glutamine synthetase (GS) at 1:250 (Abcam), anti-glial fibrillary acidic protein (GFAP) at 1:100 (Abcam), anti-microphthalmia-associated transcription factor (MITF) at 1:100 (Abcam), anti-calponin at 1:100 (Abcam), anti-von Willebrand factor (vWF) at 1:100 (EMD Millipore), anti-synaptophysin at 1:100 (EMD Millipore), and anti-postsynaptic density-95 (PSD-95) at 1:100 (EMD Millipore). The secondary antibodies used were as follows: goat anti-mouse IgG Alexa Fluor® 488 at 1:500 (Thermo Fisher Scientific), goat anti-rabbit IgG Alexa Fluor® 488 at 1:500 (Thermo Fisher Scientific), goat anti-mouse IgG Alexa Fluor® 555 at 1:500 (Thermo Fisher Scientific), and goat anti-rabbit IgG Alexa Fluor® 555 at 1:500 (Thermo Fisher Scientific).

All experiments included the following controls: primary antibody only, secondary antibody only, and no antibody.

### Western blotting

Western blotting was performed as described previously [36, 37]. Retinas were isolated from mice at various time points and homogenized in an ice-cold mixture of RIPA buffer (Beyotime, Shanghai, China) and proteinase inhibitor (Beyotime). After the protein concentration was measured using the BCA test (Beyotime), proteins were separated using 10–12% sodium dodecyl sulfate polyacrylamide gels and transferred onto polyvinylidene fluoride membranes. The membranes were blocked with Tris-buffered saline (12.5 mM Tris–HCl, pH 7.6, 75 mM NaCl) containing 5% fat-free milk and 0.1% Tween 20 for 1 hour at room temperature. The membranes were then incubated with the primary antibody at 4 °C overnight and with a peroxidase-conjugated secondary antibody for 1 hour at room temperature. Chemiluminescent results were obtained using the Odyssey infrared imaging system with the Odyssey Application software V1.2.15 (LI-COR Biosciences, Lincoln, NE, USA) and analyzed using ImageJ software (National Institutes of Health, Bethesda, MD, USA). The relative level of recoverin, rhodopsin, synaptophysin, PSD-95, and c-kit were determined via normalization against β-actin.

The primary antibodies used were as follows: anti-recoverin at 1:1000 (EMD Millipore), anti-rhodopsin at 1:1000 (Abcam), anti-synaptophysin at 1:1000 (EMD Millipore), anti-PSD-95 at 1:500 (EMD Millipore), anti-c-kit at 1:1000 (Cell Signaling Technology), and anti-β-actin at 1:1000 (Abcam). The secondary antibodies used were as follows: peroxidase-conjugated goat anti-mouse IgG at 1:2000 (Beyotime) and peroxidase-conjugated goat anti-rabbit IgG at 1:2000 (Beyotime).

### Cell labeling

The labeling procedure for quantum nanocrystal dots (QDs) before transplantation was performed according to the instructions for the Qtracker® Cell Labeling Kit (Thermo Fisher Scientific). Qtracker® component A and component B (1:1) were mixed and incubated at room temperature for 5 min and then added into the growth medium to prepare a 10 nM working concentration of labeling solution. Cells were incubated with the labeling solution at 37 °C for 60 min. After being washed with PBS, cells were resuspended with PBS at 2 × 10^5^ cells/μl and kept on ice prior to transplantation.

### Cell transplantation

At PND 7, rd1 mice were anesthetized with 1.5–2% isoflurane. Cells were injected using a sharp 33-gauge needle (Hamilton Storage, Franklin, MA, USA) that was inserted tangentially through the sclera and into the subretinal space. In total, 1 × 10^5^ cells (0.5 μl per injection) were slowly injected over the course of at least 30 seconds.

For the mice to be used in the flash-electroretinogram (F-ERG) test, one eye was transplanted with cells, while the other eye was injected with PBS as a control. For the mice used in the light/dark transition test, both eyes were injected with cells. The control mice were injected with PBS in both eyes, and uninjected age-matched mice were used as controls.

### F-ERG recording

The ERG recording procedures were performed as described previously [34]. Mice were tested at 4 weeks and 8 weeks (*n* = 5 eyes for each time point). Transplanted eyes received cells, while the contralateral control eye received an identical sham cell injection containing PBS instead of cells. Mice were adapted to darkness overnight, and all of the recording procedures were performed under dim red light. After anesthesia with 1.5–2% isoflurane, mice were kept warm on a heating pad and maintained at 37 °C. Pupils were dilated with tropicamide and phenylephrine eye drops (Santen Pharmaceutical, Osaka, Japan). Electrodes were placed at the cornea as recording electrodes, inserted under the skin of the angulus oculi as reference electrodes, and inserted under the skin of the tail as grounding electrodes. Single flash recordings were obtained at the light intensities of 0.5 log_10_ (cd s/m^2^) and acquired using Reti-scan system (Roland Consult, Havel, Germany). The a-wave and b-wave amplitudes were analyzed to compare the treated eyes with contralateral eyes.

### Light/dark transition test

The light/dark transition test was performed as described previously [38]. The mice received bilateral cell transplantation, or bilateral PBS injection for control. The light/dark box (45 cm × 30 cm × 40 cm) consisted of a light chamber (30 cm × 30 cm × 40 cm) and a dark chamber (15 cm × 30 cm × 40 cm) connected with a 10 cm × 10 cm door in the middle. The mice were dark-adapted overnight and stayed in the dark chamber for 2 min before the test without any light stimulus. After the habituation period, the mice were allowed to explore the both chambers for 5 min. The test field was lit at 300 lux by a tungsten filament bulb positioned over the center of the light chamber. All of the mice were test naïve (only one test per mouse). The length of the time spent in the light area was video-recorded and calculated. Entering into a chamber was defined as four paws having crossed the connecting door.

### Statistical analysis

Statistical analyses were performed using SPSS 22.0. Data are presented as the mean ± standard deviation (SD). For comparisons among groups, a one-way ANOVA followed by Fisher’s protected least-significant difference post test was used for multiple comparisons. Differences were accepted as significant at *P* < 0.05.

## Results

### Characterization of c-kit^+^/SSEA1^−^ cells isolated from the mouse eye wall

**Fig. 1:**
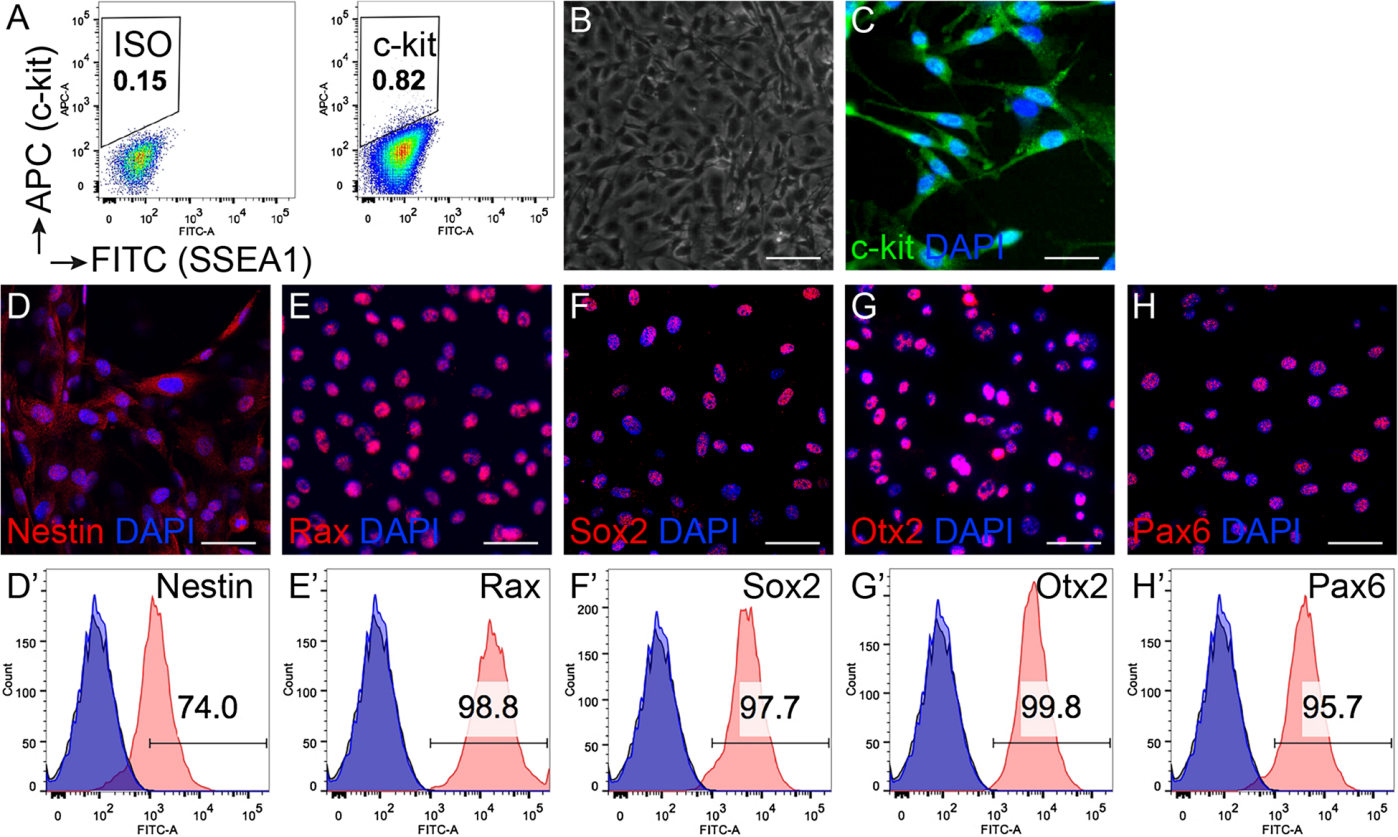
Progenitor characteristics of mouse eye-wall c-kit^+^/SSEA1^−^ cells. (**A**) Representative flow cytometry plots showing the percentage of c-kit-positive SSEA1-negative cells. Gating was established based on cells stained with isotype-matched APC and FITC antibodies (*ISO*; *left panel*). Representative flow cytometry plots showed that c-kit^+^/SSEA1^−^ cells represented approximately 0.82% of the total population (*right panel*). (**B**) Phase-contrast image of representative c-kit^+^/SSEA1^−^ cells in culture. (**C**) Representative image of immunofluorescence staining for c-kit^+^ (*green*) with 4′,6-diamidino-2-phenylindole (*DAPI*; *blue*). (**D–H**) Representative images of immunofluorescence for RPC markers (*red*) and DAPI (*blue*), showing that cells express Nestin (**D**), retina homeobox protein Rx (*Rax*; **E**), SRY-box 2 (*Sox2*; **F**), Orthodenticle Homeobox 2 (*Otx2*; **G**), and paired box protein 6 (*Pax6*; **H**). (**D′–H′**) Representative flow cytometry plots showing expression in the FITC channel for Nestin (74%; **D′**), Rax (98.8%; **E′**), Sox2 (97.7%; **F′**), Otx2 (99.8%; **G′**), and Pax6 (95.7%; **H′**). *Scale bars* represent 50 μm (Color figure online)

Progenitor cells were harvested from the eye wall of PND 1 mice. After one passage expansion, FACS was used to isolate c-kit^+^/SSEA1^−^ cells. The percentage of c-kit^+^/SSEA1^−^ cells was usually approximately 1% (representative image in Fig. 1A and more supporting data in Additional file 1: Figure S1). Phase-contrast imaging showed that the c-kit^+^/SSEA1^−^ cells grew in the dishes (Fig. 1B) and these cells expressed c-kit (Fig. 1C). After proliferation in vitro, we detected that the eye-wall c-kit^+^/SSEA1^−^ progenitor cells expressed the markers of retinal progenitor cells (RPCs), including nestin, Rax, Sox2, Otx2, and Pax6, by immunofluorescence staining (Fig. 1D–H) and flow cytometry (Fig. 1D′–H′).

**Fig. 2:**
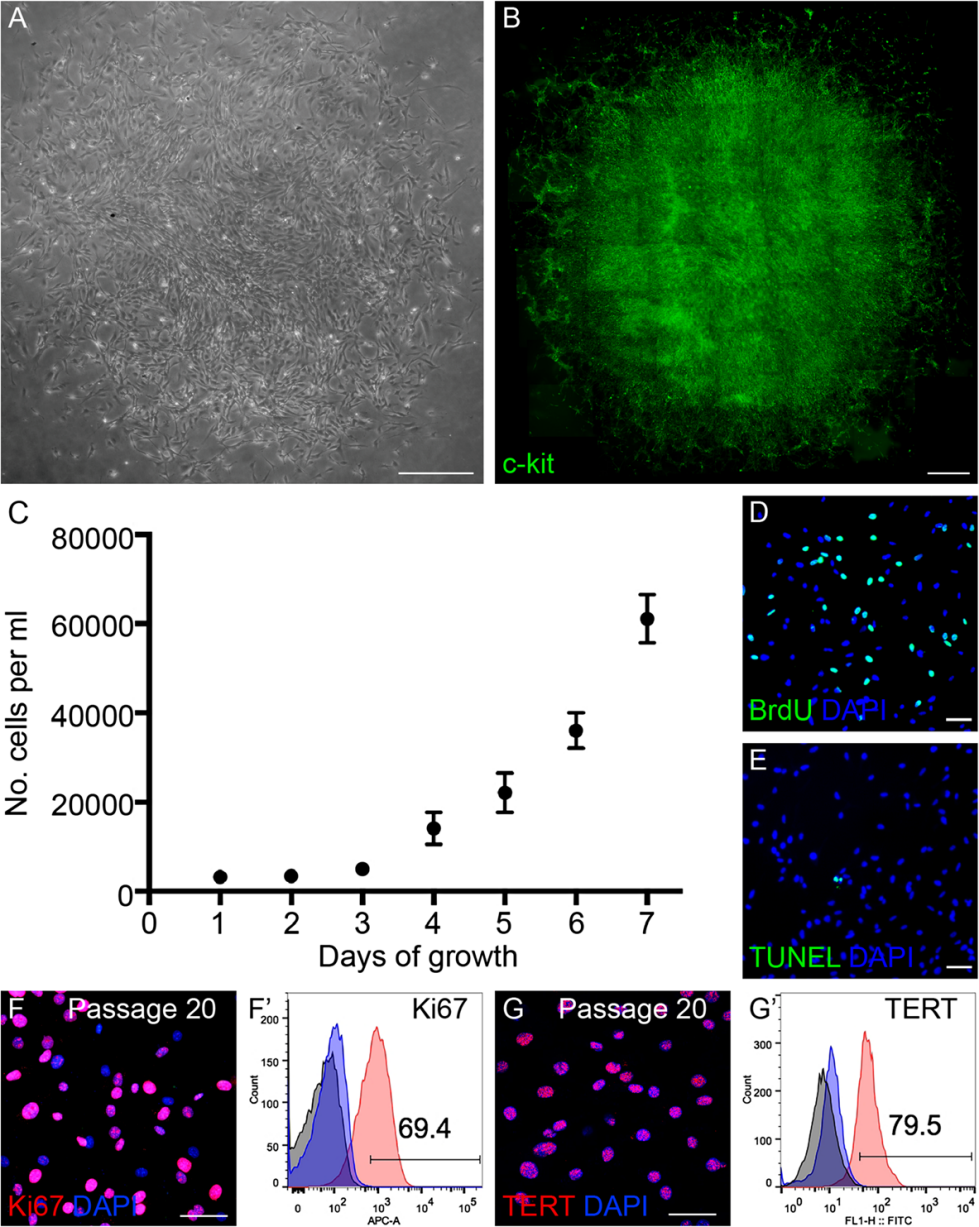
Self-renewal capacity of mouse eye-wall c-kit^+^/SSEA1^−^ cells. (**A, B**) Phase-contrast image of a representative putative clone of c-kit^+^/SSEA1^−^ cells (**A**) and the clone with immunofluorescence staining for c-kit (*green*; **B**). (**C**) Growth curve of c-kit^+^/SSEA1^−^ cells over a 7-day period. (**D, E**) 5-Bromo-2'-deoxyuridine (*BrdU*) labeling (**D**) and terminal deoxy nucleotidyl transferase-mediated nick end labeling (*TUNEL*; **E**) staining of c-kit^+^/SSEA1^−^ cells on the 7th day showed that the cells kept in active proliferation and the level of apoptosis was quite low. (**F, G**) After 20 passages, c-kit^+^/SSEA1^−^ cells retained expression of Ki67 (**F**) and telomerase reverse transcriptase (*TERT*; **G**). (**F′, G′**) Representative flow cytometry plots showing the expression of Ki67 (69.4%; **F′**) and TERT (79.5%; **G′**). *DAPI* 4′,6-diamidino-2-phenylindole. *Scale bars* represent 400 μm (**A, B**) and 50 μm (**D–G**) (Color figure online)

We plated the cells at a low density (1 cell/60 mm^2^) to evaluate the clone-formation properties of c-kit^+^ cells based on our previous studies [33]. A clone could be formed from a single cell (Fig. 2A) and maintain its c-kit expression (Fig. 2B). The growth curve showed that c-kit^+^/SSEA1^−^ cells grew well in vitro. For the first 3 days, the cell number remained stable. From the 4th day onward, the cell number increased by 1.5–2 times compared with the earlier day (Fig. 2C). On the 7th day, the cells still actively divided (Fig. 2D) and the apoptosis was at a low level (Fig. 2E). After 20 passages, c-kit^+^ cells still maintained high percentages of cell division and TERT expression (Fig. 2F, G).

### Differentiation ability of the eye-wall c-kit^+^/SSEA1^−^ progenitor cells

**Fig. 3:**
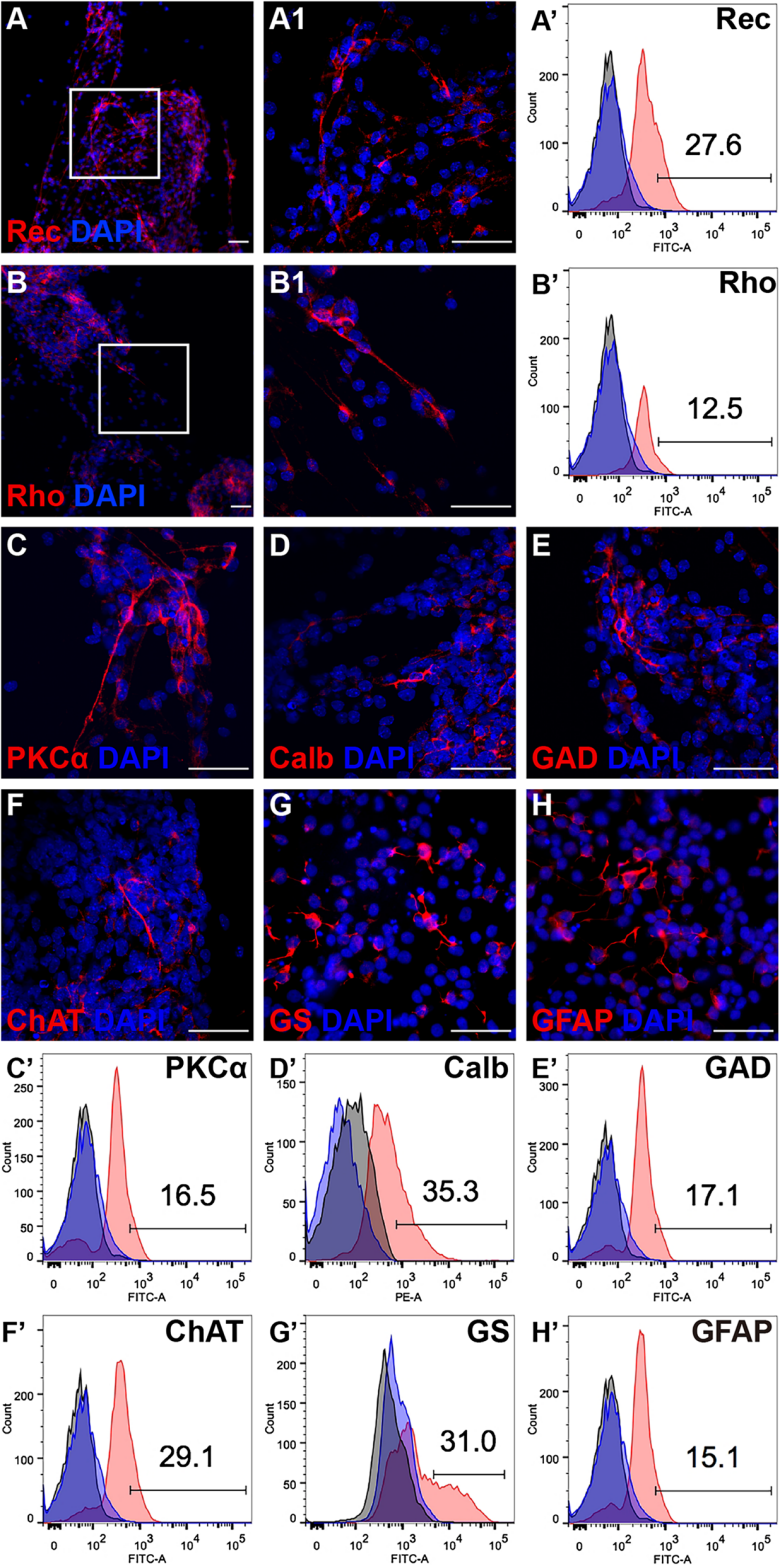
Neural retinal differentiation potential of eye-wall c-kit^+^/SSEA1^−^ progenitor cells. The cells were cultured in differentiation media for 8–10 days and were stained with markers for neurons or Müller cells and with DAPI for counting nuclei (*blue*). Representative images showing cells positive for Recoverin (*Rec*; **A**), Rhodopsin (*Rho*; **B**), protein kinase C alpha (*PKCα*; **C**), Calbindin (*Calb*; **D**), glutamate decarboxylase 65 & 67 (*GAD*; **E**), choline acetyltransferase (*ChAT*; **F**), glutamine synthetase (*GS*; **G**), and glial fibrillary acidic protein (*GFAP*; **H**). Areas in the *white boxes* in **A** and **B** are shown at higher magnification in **A1** and **B1**, respectively. Cells were harvested after differentiation for 8–10 days and were stained for markers of neurons and Müller cells, as shown in the FITC and PE channels. Representative flow cytometry plots showing the percentages of cells positive for Rec (27.6%; **A′**), Rho (12.5%; **B′**), PKCα (16.5%; **C′**), Calb (35.3%; **D′**), GAD (17.1%; **G′**), ChAT (29.1%; **F′**), GS (31%; **G′**), and GFAP (15.1%; **H′**). *DAPI* 4′,6-diamidino-2-phenylindole. *Scale bars* represent 50 μm for all images (Color figure online)

In the specific differentiation media, the eye-wall c-kit^+^/SSEA1^−^ cells differentiated into various cell types: photoreceptors, observed via staining for recoverin (Fig. 3A) and rhodopsin (Fig. 3B); bipolar cells, via staining for PKCα (Fig. 3C); horizontal cells, via staining for calbindin (Fig. 3D); amacrine cells, via staining for GAD (Fig. 3E) and ChAT (Fig. 3F); and Müller cells, via staining for GS (Fig. 3G) and GFAP (Fig. 3H). The differentiation ratio of these cell types were confirmed with flow cytometric analysis (Fig. 3A′–3H′). In photoreceptor differentiation medium, approximately 27.6% of cells expressed recoverin (Fig. 3A′) and 12.5% expressed rhodopsin (Fig. 3B′). In horizontal cell differentiation medium, approximately 35.3% of cells expressed calbindin (Fig. 3D′). For specific differentiation to amacrine cells, approximately 17.1% and 29.1% of cells expressed GAD (Fig. 3E′) and ChAT (Fig. 3F′), respectively. When the eye-wall c-kit^+^/SSEA1^−^ cells were cultured in medium without specific differentiation stimuli, approximately 16.5% of cells expressed PKCα (Fig. 3C′), 31% of cells expressed GS (Fig. 3G′), and 15.1% of cells expressed GFAP (Fig. 3H′).

**Fig. 4:**
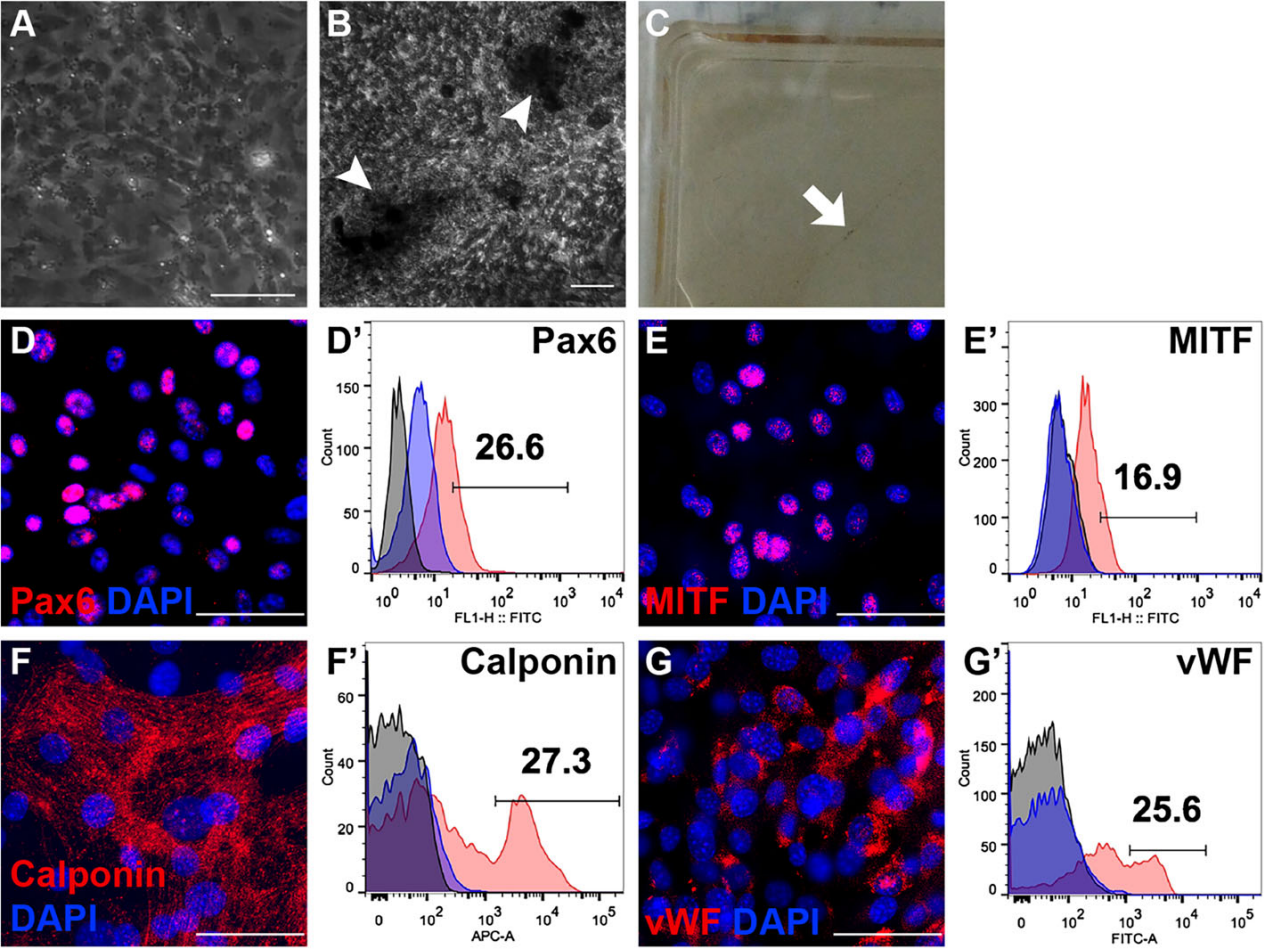
Transdifferentiation capability of eye-wall c-kit^+^/SSEA1^−^ progenitor cells. (**A**) Day 1 in which the medium of c-kit^+^/SSEA1^−^ cells was switched to the RPE differentiation medium. (**B**) Pigment (*arrowhead*) appeared after 4–8 weeks. (**C**) Pigment (*arrow*) could be seen in the dish. Representative immunostaining images showing cells positive for paired box protein 6 (*Pax6*; **D**), microphthalmia-associated transcription factor (*MITF*; **E**), Calponin (**F**), and von Willebrand factor (*vWF*; **G**). Differentiated cells were stained for markers shown in the FITC and APC channels. Representative flow cytometry plots showing the percentages of cells positive for Pax6 (26.6%; **D′**), MITF (16.9%; **E′**), Calponin (27.3%; **F′**), and vWF (25.6%; **G′**). *DAPI* 4′,6-diamidino-2-phenylindole. *Scale bars* represent 50 μm for all the images (Color figure online)

When the eye-wall c-kit^+^/SSEA1^−^ cells were placed in nonselective differentiation medium for RPE cells, pigment appeared at 4–8 weeks (Fig. 4B, C). Pax6 and MITF are expressed in the early stage of RPE cells. Immunostaining and flow cytometry showed that differentiated cells expressed Pax6 (26.6%; Fig. 4D, D′) and MITF (16.9%; Fig. 4E, E′). In addition to the major cell types of the retina, we assessed whether the eye-wall c-kit^+^/SSEA1^−^ cells could transdifferentiate into smooth muscle cells and endothelial cells in culture. The proportions of cells that differentiated into smooth muscle cells (calponin; Fig. 4F, F′) and endothelial cells (vWF; Fig. 4G, G′) were 27.3% and 25.6%, respectively.

### Photoreceptor differentiation of mouse eye-wall c-kit^+^/SSEA1^−^ cells in the retina of rd1 mice

During the first week after birth, c-kit expression in wild-type mice and rd1 mice did not show significant difference. At PND 8, the expression of c-kit in rd1 was increased compared with age-matched wild-type mice, and it immediately declined thereafter (Additional file 2: Figure S2). As the retina of rd1 mouse begins to degenerate at PND 8–10 [39–43], we transplanted the eye-wall c-kit^+^/SSEA1^−^ cells on PND 7, immediately before the initiation of degeneration.

**Fig. 5:**
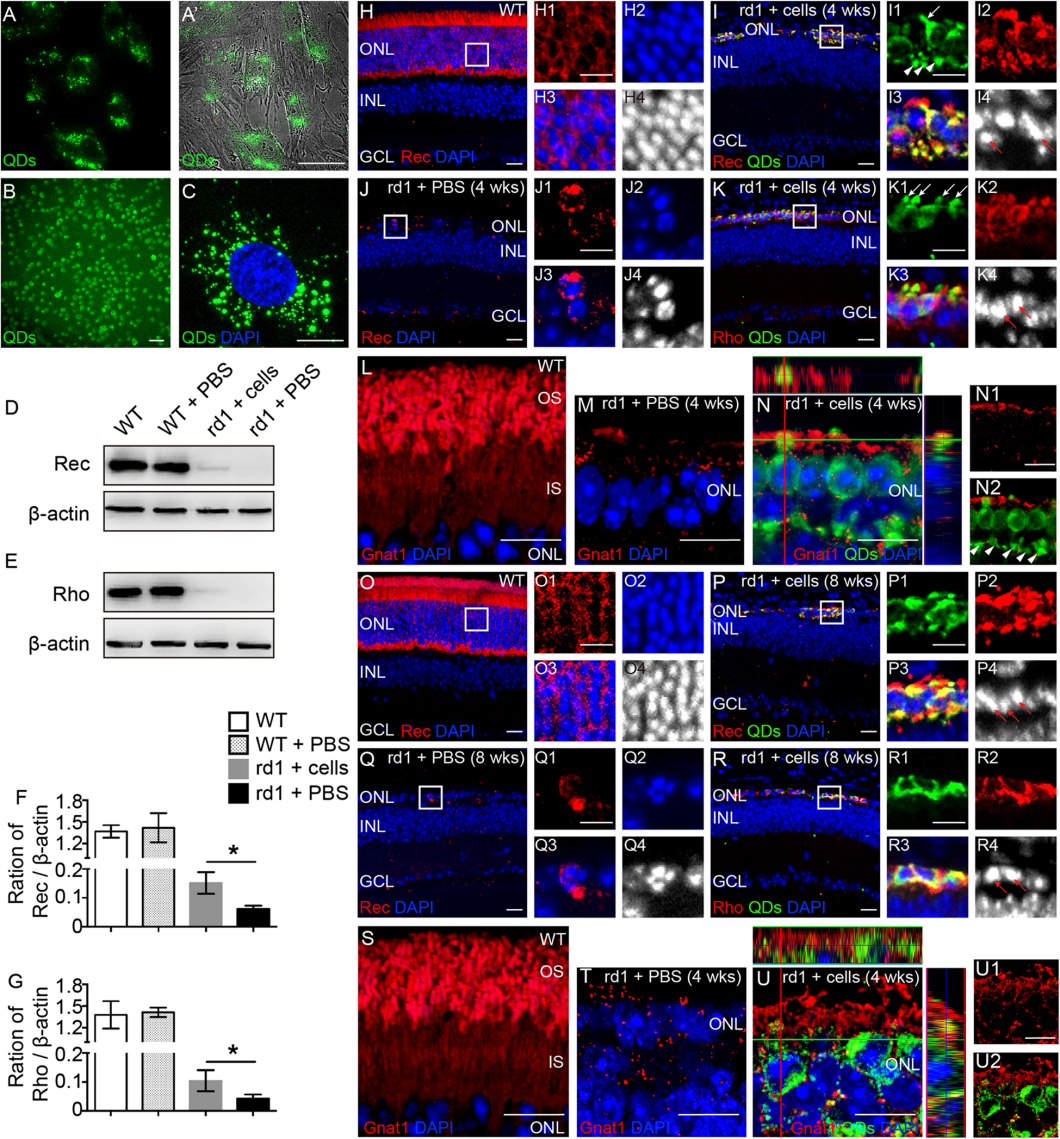
Differentiation of engrafted eye-wall c-kit^+^/SSEA1^−^ progenitor cells (*green*) in the retinas of rd1 mice. (**A–D**) Cells were labeled using quantum nanocrystal dots (*QDs*) before transplantation. Cells cultured in a dish and incubated with QDs labeled with green fluorescence (**A**) and merged with phase contrast (**A′**). Under digestion conditions, a high rate of labeling was observed (**B**). After fixation, QDs in the cells could still be seen clearly (**C**). Representative western blot bands of recoverin (*Rec*) (**D**) and rhodopsin (*Rho*) (**E**) versus β-actin at 4 weeks after transplantation. The blots were then quantitated for expression of Rec (**F**) and Rho (**G**), corrected for β-actin, and plotted as the mean expression ± SD (*n* = 3 for each time point, *P < 0.05). Representative images of rd1 mice injected with c-kit^+^/SSEA1^−^ cells at 4 weeks (**I, K, N**) and 8 weeks (**P, R, U**) after transplantation and age-matched control mice including wild-type (*WT*) mice (**H, L, O, S**) and rd1 mice injected with phosphate-buffered saline (*PBS*; **J, M, Q, T**). The outer nuclear layer (*ONL*) of WT mice expressed Rec generally (**H, O**) and formed the structure of inner segment (*IS*) and outer segment (*OS*; **L, S**); there were few Rec-expressing photoreceptors remaining in the PBS control group and IS/OS was hardly observed (**J, M**, 4 weeks; **Q, T**, 8 weeks). Immunohistochemical detection of c-kit^+^/SSEA1^−^ cell-derived photoreceptors expressing Rec (**I**) and Rho (**K**) with rod a-transducin (*Gnat1*)-positive OS-like structure (**N**) at 4 weeks and 8 weeks (**P, R, U**) post transplantation. Area in the *white boxes* is shown at higher magnification. *White arrow*, engrafted cells could form IS/OS-like structures; *red arrow*, engrafted cells had condensed nuclei, similar to normal rod photoreceptors; *white arrowhead*, engrafted cells could develop synaptic button-like structures. *DAPI* 4′,6-diamidino-2-phenylindole, *INL* inner nuclear layer, *GCL* ganglion cell layer. *Scale bars* represent 5 μm (**C**), 10 μm (**H1–H4, I1–I4, J1–J4, K1–K4, L, M, N, N1–N2, O1–O4, P1–P4, Q1–Q4, R1–R4, S, T, U, U1–U2**), and 20 μm (**A, B, H–K, O–R**) (Color figure online)

The eye-wall c-kit^+^/SSEA1^−^ cells were prelabeled with QDs. The green fluorescence linked to the QDs could be detected when cells were still attached to the dish (Fig. 5A, A′), floated after digestion (Fig. 5B), and stained after fixation (Fig. 5C).

Western blot assays showed that, at 4 weeks after transplantation, the retina with injected cells showed faint bands representing significantly higher expression levels for recoverin (Fig. 5D, F) and rhodopsin (Fig. 5E, G) than in the PBS-injection control.

At 4 weeks post transplantation, most of the transplanted cells were located in the outer nuclear layer (ONL). Confocal images showed that grafted cells with green fluorescence subsequently expressed recoverin (Fig. 5I) and rhodopsin (Fig. 5K; red fluorescence). This fluorescence demonstrated that c-kit^+^/SSEA1^−^ cells differentiated into photoreceptors (merged as yellow fluorescence; Fig. 5I3, K3), and some of these c-kit^+^/SSEA1^−^ cell-derived photoreceptor-like cells exhibited the morphology of the inner segment (IS)/outer segment (OS; Fig. 5I1, K1, white arrows) and had condensed nuclei (Fig. 5I4, K4, red arrows), which are typical structural characteristics of photoreceptors. Furthermore, in transplanted donor cells, we found immunoreactivity against rod a-transducin (Gnat1), a protein essential for rod phototransduction and normally localized in the OS of rods. As further evidence of maturation, donor cells expressed mature rod-specific marker Gnat1 4 weeks after transplantation (Fig. 5N). Also, engrafted cells could develop synaptic button-like structures (Fig. 5I1, N2, white arrowhead). At 8 weeks after transplantation, the immunostaining images showed data consistent with 4 weeks post transplantation (Fig. 5P, R, U). For the time-matched PBS injection group, there were few recoverin-expressing cells remaining and the IS/OS was hardly observed (Fig. 5J, M, 4 weeks; Fig. 5Q, T, 8 weeks).

### Synapse formation between engrafted c-kit^+^/SSEA1^−^ cell-derived photoreceptors and host bipolar cells

**Fig. 6:**
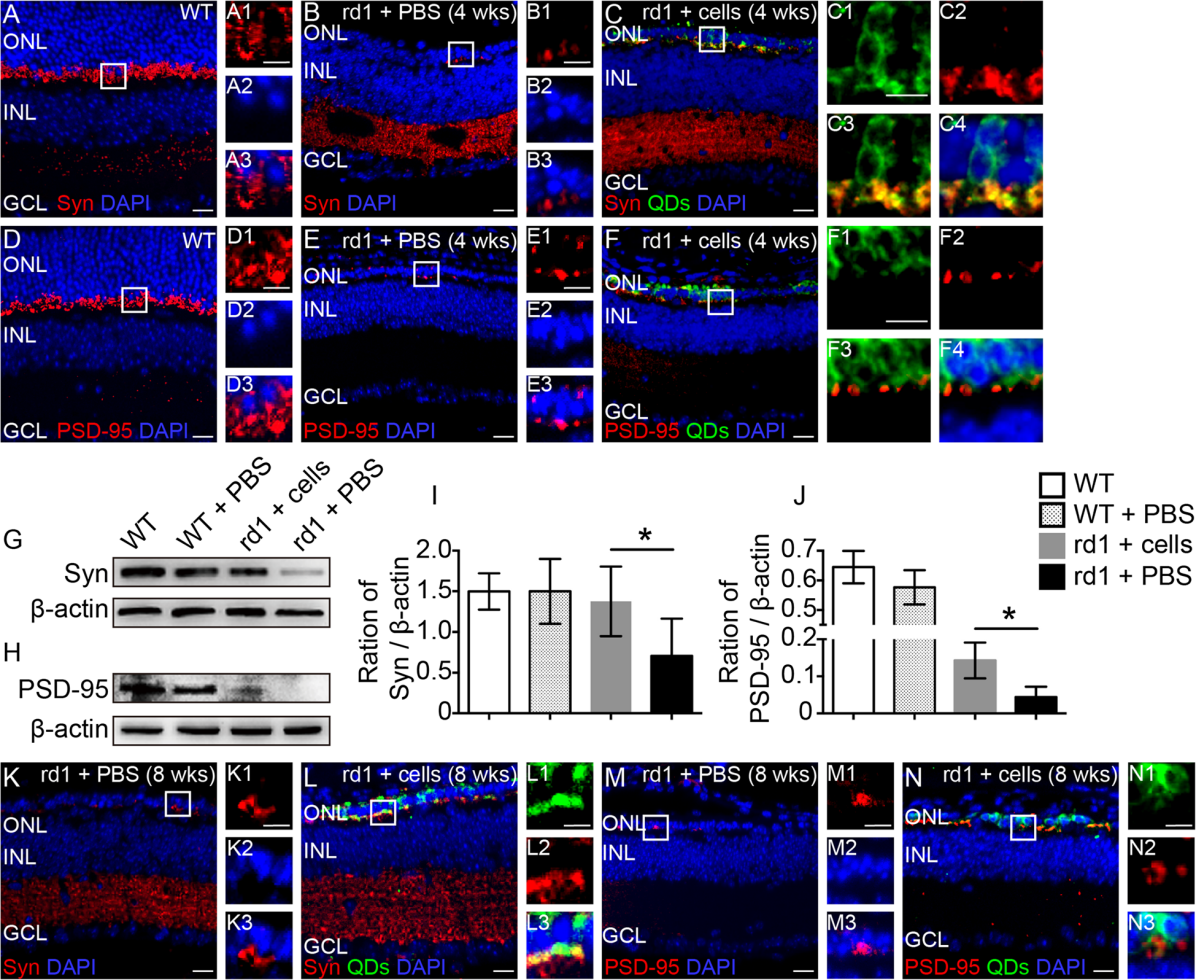
Synapse formation between engrafted eye-wall c-kit^+^/SSEA1^−^ cell-derived photoreceptors and the bipolar cells of rd1 mice. Representative images of rd1 mice injected with c-kit^+^/SSEA1^−^ cells at 4 weeks (**C, F**) and 8 weeks (**L, N**) after transplantation and of age-matched control mice including wild-type (*WT*) mice (**A, D**) and rd1 mice injected with phosphate-buffered saline (*PBS*; **B, E, K, M**). WT mice expressed synaptophysin (*Syn*) and postsynaptic density-95 (*PSD-95*) in the OPL (**D**); the PBS control group showed much lower levels of these proteins (**B, E**, 4 weeks; **K, M**, 8 weeks). Syn-immunoreactive puncta were observed on the cell membrane of engrafted c-kit^+^/SSEA1^−^ cells at 4 weeks (**C**) and 8 weeks (**L**) post transplantation. PSD-95-positive postsynaptic terminals contacted incorporated donor cell-derived photoreceptors at 4 weeks (**F**) and 8 weeks (**N**) post transplantation. Representative western blot bands of Syn versus β-actin (**G**) and PSD-95 versus β-actin (**H**) at 4 weeks after transplantation. The blots were then quantitated for expression of Syn (**I**) and PSD-95 (**J**), corrected for β-actin, and plotted as the mean expression ± SD (*n* = 3 for each time point, *P < 0.05). *DAPI* 4′,6-diamidino-2-phenylindole, *ONL* outer nuclear layer, *INL* inner nuclear layer, *GCL* ganglion cell layer, *QD* quantum nanocrystal dot. *Scale bars* represent 10 μm (**A1–A3, B1–B3, C1–C4, D1–D3, E1–E3, F1–F4, K1–K3, L1–L3, M1–M3, N1–N3**) and 20 μm (**A–F, K–N**) (Color figure online)

In wild-type mice, synaptophysin (Fig. 6A) and PSD-95 (Fig. 6D) are usually expressed in the outer plexiform layer (OPL), where photoreceptors make synaptic connections with bipolar cells. Synaptophysin is located in the presynaptic membrane, in photoreceptors, and PSD-95 is located in the postsynaptic membrane, in bipolar cells. Meanwhile, synaptophysin is also expressed in the inner plexiform layer between bipolar cells and ganglion cells. At 4 and 8 weeks post transplantation, the graft–host interface between c-kit^+^/SSEA1^−^ cell-derived photoreceptors (green fluorescence) and host retina expressed synaptophysin (red fluorescence; Fig. 6C, L) and PSD-95 (red fluorescence; Fig. 6F, N). In the merged image, synaptophysin colocalized with engrafted cells (yellow fluorescence; Fig. 6C4, L3), while PSD-95 did not colocalize with the green cells (Fig. 6F4, N3), which implied that synaptophysin was expressed on the terminals of c-kit^+^/SSEA1^−^ cell-derived photoreceptors and that PSD-95 was expressed on the downstream bipolar cells of the rd1 mice. Western blot assay demonstrated that at 4 weeks after transplantation, the levels of synaptophysin (Fig. 6G, I) and PSD-95 (Fig. 6H, J) in the retinas of c-kit^+^/SSEA1^−^ cell-transplanted rd1 mice were significantly higher than in the PBS injection control group, which indicated that cell transplantation improved neural plasticity in the retinas of rd1 mice.

### Eye-wall c-kit^+^/SSEA1^−^ cell transplantation alleviated morphological abnormalities of the inner retina of rd1 mice

**Fig. 7:**
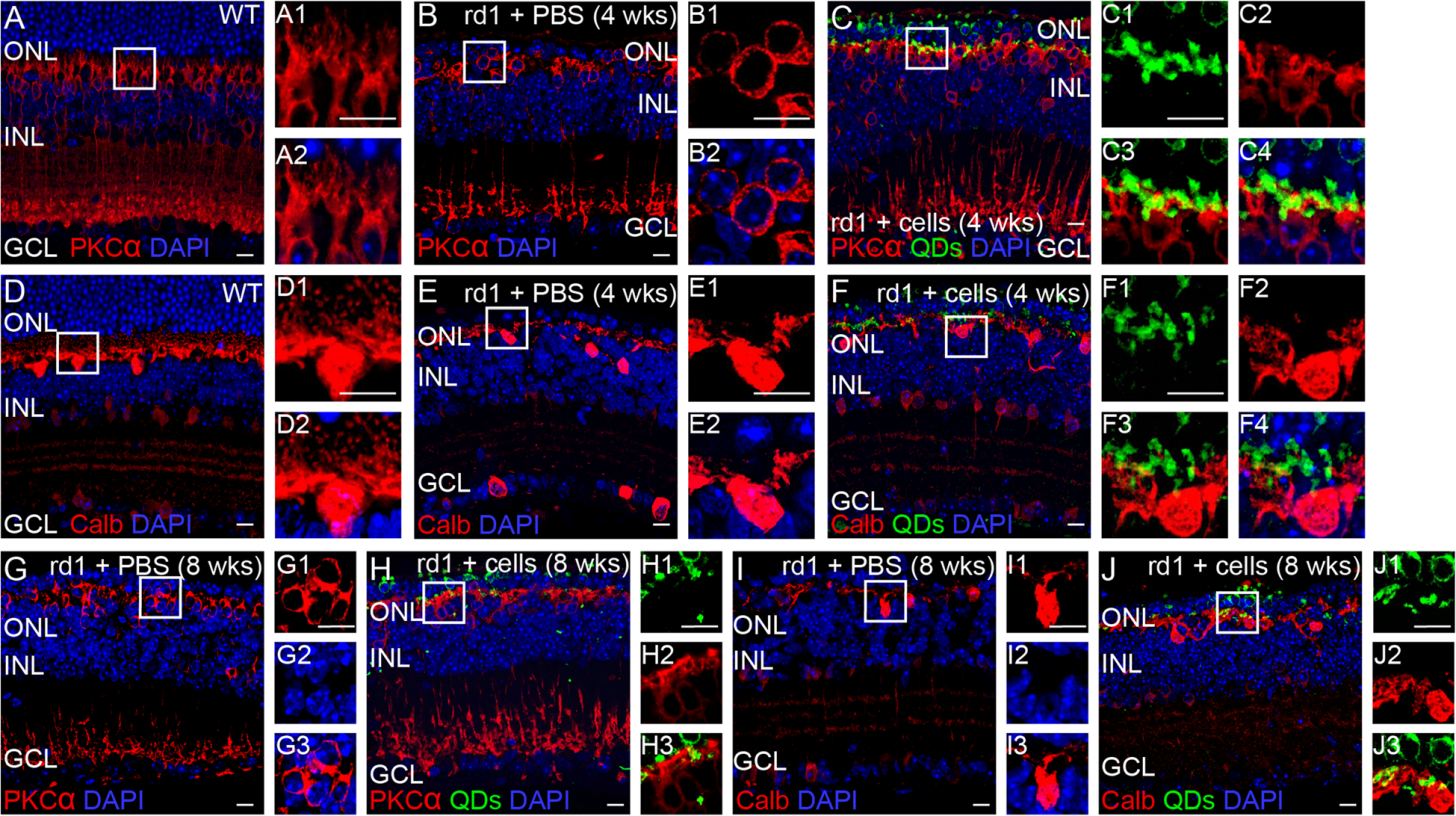
Morphological modifications in the inner retina of the rd1 mice after eye-wall c-kit^+^/SSEA1^−^ cell transplantation. Representative images of rd1 mice injected with c-kit^+^/SSEA1^−^ cells at 4 weeks (**C, F**) and 8 weeks (**H, J**) after transplantation and age-matched control mice including wild-type (*WT*) mice (**A, D**) and rd1 mice injected with phosphate-buffered saline (*PBS*; **B, E, G, I**). WT mice showed protein kinase C alpha (*PKCα*)-positive bipolar cells (**A**) and Calbindin (*Calb*)-positive horizontal cells (**D**) in the inner nuclear layer (*INL*). The PBS control group showed that the projections of PKCα-positive bipolar cells and Calb-positive horizontal cells appeared sparse, short, and disorganized (**B, E**, 4 weeks; **G, I**, 8 weeks). More PKCα-immunoreactive projections were observed between engrafted c-kit^+^/SSEA1^−^ cells and host bipolar cells at 4 weeks (**C**) and 8 weeks (**H**) post transplantation. Calb-positive fine-size processes remained when connecting incorporated donor cell-derived photoreceptors at 4 weeks (**F**) and 8 weeks (**J**) post transplantation. *DAPI* 4′,6-diamidino-2-phenylindole, *ONL* outer nuclear layer, *GCL* ganglion cell layer, *QD* quantum nanocrystal dot. *Scale bars* represent 10 μm (Color figure online)

The dendritic arbors of PKCα-positive rod bipolar cells in the rd1 mice were shorter and spatially disordered (Fig. 7B, G), compared with age-matched wild-type mice (Fig. 7A). After the eye-wall c-kit^+^/SSEA1^−^ progenitor cell transplantation, some of the PKCα-positive bipolar cells kept bushy dendrites which oriented to the engrafted cells, especially at the transplantation area (Fig. 7C, 4 weeks; Fig. 7H, 8 weeks).

Compared with wild-type mice (Fig. 7D), bodies of calbindin-positive horizontal cells in rd1 mice were still arranged regularly while the axonal complexes were very poorly organized in the OPL. Large-size processes remained in the OPL of rd1 mice while fine-size processes were lost (Fig. 7E, I). After cell transplantation, some fine-size processes of horizontal cells were retained especially in the engrafted c-kit^+^/SSEA1^−^ cell area (Fig. 7F, 4 weeks; Fig. 7J, 8 weeks).

### Engrafted eye-wall c-kit^+^/SSEA1^−^ cells contributed to the restoration of visual function in rd1 mice

**Fig. 8:**
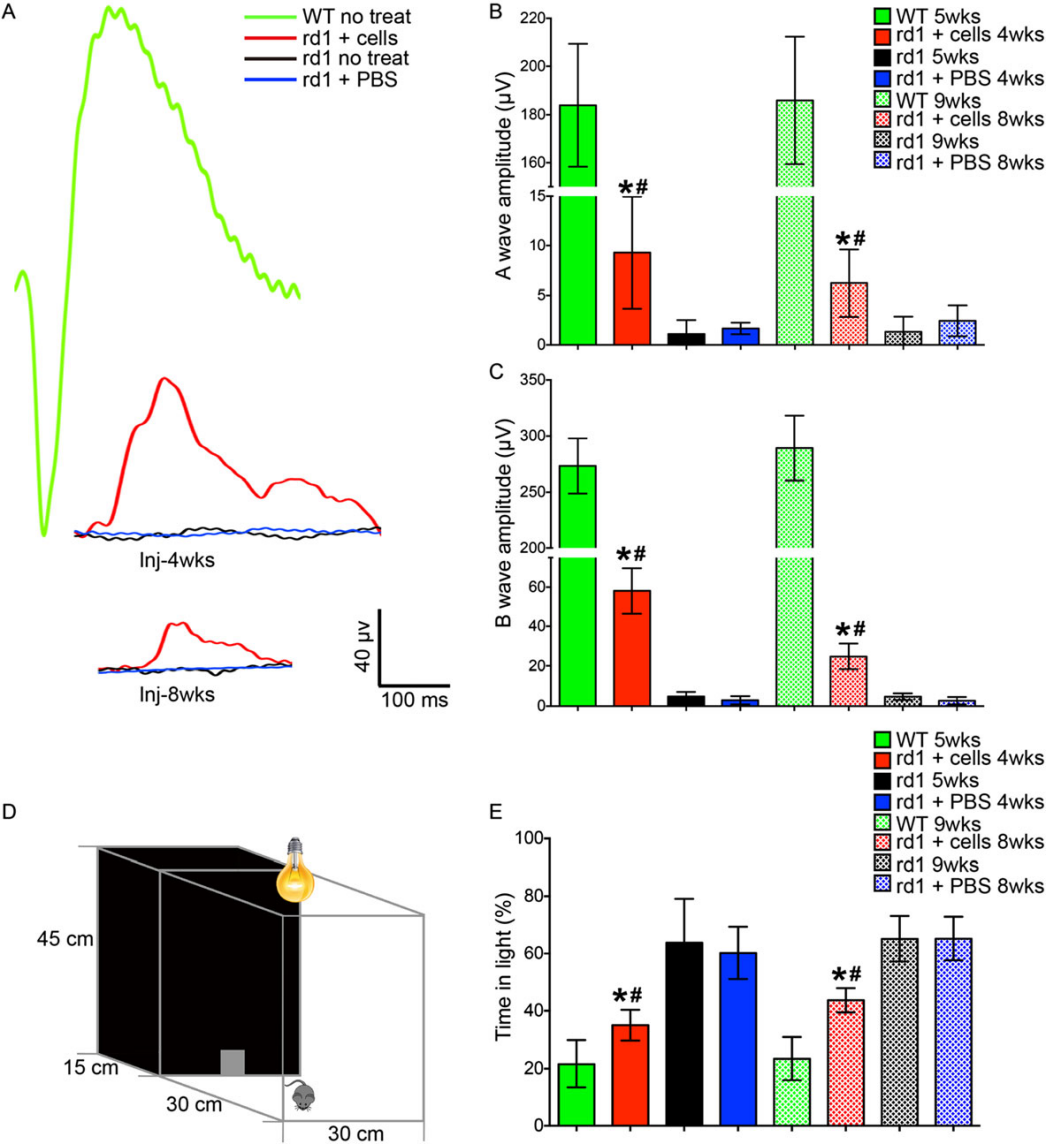
Functional improvement of rd1 mice transplanted with eye-wall c-kit^+^/SSEA1^−^ progenitor cells. F-ERG tests and light/dark transition tests were performed at 4 and 8 weeks post transplantation. **A** F-ERG tests showed that the visual acuity of the rd1 mice were improved in each group with transplantation of c-kit^+^/SSEA1^−^ cells compared with corresponding controls (phosphate-buffered saline (*PBS*)-injected and uninjected mice). **B**, **C** Transplanted eyes exhibit significantly increased a-wave (**B**) and b-wave (**C**) amplitudes after light flash compared with control eyes, though they did not reach the amplitude exhibited by normal wild-type mouse eyes. **D** The light/dark transition test box consisted of a dark compartment (one-third of the floor area) and a larger illuminated compartment (two-thirds). A small opening located at floor level in the center of the dividing wall allowed mice to freely move between the light and dark chambers. (**E**) Time spent in the light area by four groups of mice. The c-kit^+^/SSEA1^−^ cell-treated rd1 mice showed a behavioral aversion to light: they spent significantly more time in the dark chamber than either PBS-injected or uninjected rd1 mice. Data shown as the mean ± SD. *P < 0.05 vs PBS injection controls, #*P* < 0.05 vs uninjected controls. *WT* wild type (Color figure online)

F-ERG tests and the light/dark transition test were performed 4 and 8 weeks after cell transplantation. The F-ERG revealed that rd1 mice that received PBS injections at PND 7 showed no difference from uninjected rd1 mice in the amplitude of the a-wave and b-wave after 4 weeks (Fig. 8A), whereas c-kit^+^/SSEA1^−^ cell transplantation significantly increased the a-wave amplitude (Fig. 8B) in rd1 mice to 9.30 ± 5.65 and 6.21 ± 3.40 μV and the b-wave amplitude (Fig. 8C) in rd1 mice to 58.2 ± 11.5 and 24.8 ± 6.51 μV (mean ± SD, *n* = 5; *P* < 0.05) after 4 weeks and 8 weeks, respectively.

In the light/dark transition test, mice were placed in the apparatus shown in Fig. 8D for a total of 5 min. Wild-type mice age-matched with mice at 4 and 8 weeks post transplantation spent 21.6 ± 8.23% and 23.4 ± 7.47%, respectively (mean ± SD, *n* = 4 for each group) in the light field, whereas rd1 mice treated with PBS spent 60.2 ± 9.17% (4 weeks, *n* = 4) and 65.2 ± 7.66% (8 weeks, *n* = 4) in the light area. c-kit^+^/SSEA1^−^ cell transplantation significantly decreased the duration spent in the light field to 35.0 ± 5.35% (*n* = 4) at 4 weeks after cell transplantation (*P* < 0.05). At 8 weeks after cell transplantation, the c-kit^+^/SSEA1^−^ cell-transplanted rd1 mice spent 43.7 ± 4.24% of the time in the light field (*n* = 4), a marked decrease compared with the PBS-treated and untreated rd1 mice (*P* < 0.05; Fig. 8E).

## Discussion

In the present study, we used FACS to isolate the c-kit^+^/SSEA1^−^ subpopulation of cells from the eye walls of newborn mice. Our results showed that these c-kit^+^/SSEA1^–^ cells expressed RPC markers, retained the capacity for cell division, and continued to express high levels of TERT after 20 passages. After transplantation into the subretinal space of rd1 mice, c-kit^+^/SSEA1^−^ cells differentiated into photoreceptors and increased the overall levels of rhodopsin and recoverin. Our data indicated that synapses had formed between engrafted c-kit^+^/SSEA1^−^ cell-derived photoreceptors and host bipolar cells and that neural plasticity had been markedly improved, ameliorating the morphological abnormalities of the INL neurons and the degeneration of visual function in rd1 mice significantly.

Retinal degeneration is caused by the progressive loss of the sensory cells of the retina, the photoreceptors, and it accounts for approximately 50% of all cases of blindness in the developed world [1–3]. Treatment efforts include attempts to replace damaged cells by transplantation and strategies for reactivating endogenous stem cell populations to generate new photoreceptors. To date, the efficiency of reactivation and the potential of the newly generated cells are low and are insufficient for the widespread repair of the mature mammalian eye after injury or disease [44–46]. As an alternative, in vitro cell culture can serve as a source of donor cells for cell replacement therapies.

During the early stages after cell transplantation, transplanted stem/progenitor cells release growth factors such as BDNF and NGF to increase the survival and function of the remaining structure [47, 48]. Meanwhile, transplanted cells might promote immunomodulation to suppress microglial activation [36]. They can also release immunoregulatory cytokines and chemokines and express immune-relevant receptors to alleviate the inflammatory response [49]. This bystander neuroprotection by neurotrophic support and/or immunomodulation plays a fundamental role in the therapeutic efficacy of stem cells at early stages. Regarding mechanisms of long-term stem cell-mediated therapy, transplanted cells might migrate from the transplantation site to the ONL, differentiate into photoreceptors, form new synaptic connections to host retinal neurons, and integrate into retinal circuits. The donor cells and their interactions with the recipient retinal microenvironment determine the outcome of the integration.

One major challenge for the recent studies has been to identify the appropriate donor cell population. Several groups have therefore examined the therapeutic effects of cells isolated from embryonic retinas followed by various expansion and differentiation protocols [50, 51]. However, these immature cells failed to integrate into the retina. Nrl^+^ postmitotic photoreceptor precursor cells labeled using green fluorescent protein (GFP) were then demonstrated to be able to correctly integrate into the retina in both the immature, developmental environment and the adult environment [19, 20, 52]. However, the stage equivalent to these precursors occurs early in the second trimester of human gestation. Obvious ethical concerns and extremely limited supply make these cells difficult to use clinically on a large scale. Relying on a three-dimensional differentiation protocol, photoreceptor precursors harvested from three-dimensional retinal cups may be safer than the photoreceptor precursors differentiated directly from embryonic stem cells (ESC) because of the organ-specific character [21]. However, this isolation procedure still depends on the genetic modification model to obtain a rhodopsin/GFP^+^ population, which will not be easy to use in the clinic. In summary, these findings demonstrate that integration is feasible if the correct stage cell is provided. However, this is not a necessary condition for integration. Another study showed that cells isolated from adult mouse retina (4–8 weeks old), forming a heterogeneous pool, could also morphologically integrate into the recipient retina [34]. Whether using retina-isolated cells or ESC-derived cells, the isolation protocol is more effective when a suitable surface marker is used to purify RPCs. In our present study, the eye-wall c-kit^+^/SSEA1^−^ cells were isolated after birth, and no tumor formation was observed in any of the experiments. Furthermore, c-kit^+^/SSEA1^−^ cells were capable of integrating into the recipient retina and permitted long-term visual restoration. As a homogeneous population isolated from postnatal retina, c-kit^+^/SSEA1^−^ cells are safe, effective, and mass-producible, meet the needs of research and clinical contexts, and might be promising donor cells in the future.

In addition to appropriate donor cells, correct integration requires successful migration of transplanted cells from the transplantation site (usually the subretinal space) through the outer limiting membrane (OLM) into the ONL [16]. Two determining factors of this process have been identified: the integrity of the OLM and the extent of recipient retinal gliosis [53–56]. The OLM, via adherens junctions between the terminal processes of the Müller glial cells and the inner segments of the photoreceptors, forms a barrier and restricts the integration of transplanted photoreceptors. It has been reported that disrupting the integrity of the OLM, via pharmacological disruption or transcriptional gene silencing of the OLM-related protein ZO-1, can improve the integration efficacy [53, 55, 56]. However, these two strategies would not be ideal for clinical applications. Toxic effects of pharmacological intervention are detrimental to Müller glial cells [57]. In addition, the OLM and the RPE layer share the adherens junction protein ZO-1, making it also a less than ideal target. On the other hand, reactive gliosis of Müller glial cells leading to glial scarring can decrease retinal integration as degeneration progresses. GFAP^−/−^Vim^−/−^ mice lacking GFAP and vimentin expression show markedly reduced levels of scarring. Subsequently, migration of transplanted cells is clearly increased [54, 58]. Confusingly, gliosis may also have beneficial effects and promote the survival of transplanted cells and remaining cone photoreceptors [59]. In an rd1 RP model, rd1 mice demonstrate severe glial scarring but also show an increase in disturbances of the OLM [53]. Although rods die rapidly, dendrites of rod bipolar cells disappear significantly more slowly [60]. In our present study, the overall levels of synaptophysin and PSD-95 increased significantly, suggesting that eye-wall c-kit^+^/SSEA1^−^ cells promote structural plasticity after degeneration.

## Conclusions

In summary, our study demonstrates that c-kit^+^ eye-wall cells possess the stem cell properties of self-renewal, colony formation, and pluripotent differentiation. Engrafted c-kit^+^ eye-wall cells could differentiate to express photoreceptor markers and could integrate into the retina of a mouse model of retinal degeneration. c-kit therefore serves as a good cell marker for the selection of candidate cells for transplantation for retinal degeneration therapy.

## Additional files

Additional file 1: Figure S1. Fluorescence-activated cell sorting of c-kit^+^/SSEA1^−^ cells. C-kit^+^/SSEA1^−^ cells were isolated in vitro illustrated by flow cytometry. (PNG 810 kb)
Additional file 2: Figure S2. C-kit expression in retina of wild-type mice and rd1 mice. Western blot analysis for c-kit expression in retinas at postnatal day (PND) 1, 3, 5, 7 (upper panel), 8, 10, 12, 14 (lower panel). (PNG 769 kb)

## Abbreviations

BDNF: Brain-derived neurotrophic factor
bFGF: Basic fibroblast growth factor
BrdU: 5-Bromo-2′-deoxyuridine
ChAT: Choline acetyltransferase
CNS: Central nervous system
DAPI: 4′,6-Diamidino-2-phenylindole
DAPT: γ-Secretase inhibitor
DHA: Docosahexaenoic acid
EGF: Epidermal growth factor
ESC: Embryonic stem cells
FACS: Fluorescence-activated cell sorting
FBS: Fetal bovine serum
GAD: Glutamate decarboxylase 65 & 67
GCL: Ganglion cell layer
GFAP: Glial fibrillary acidic protein
GFP: Green fluorescent protein
GS: Glutamine synthetase
IGF-1: Insulin-like growth factor 1
INL: Inner nuclear layer
IS: Inner segment
MITF: Microphthalmia-associated transcription factor
NGF: Nerve growth factor
ONL: Outer nuclear layer
OPL: Outer plexiform layer
OS: Outer segment
Otx2: Orthodenticle homeobox 2
Pax6: Paired box protein Pax-6
PBS: Phosphate-buffered saline
PKCα: Protein kinase C alpha
PND: Postnatal day
PSD-95: Postsynaptic density-95
Rax: Homeobox protein Rx
RD: Retinal degeneration
RP: Retinitis pigmentosa
RPC: Retinal progenitor cell
RPE: Retinal pigment epithelium
SD: Standard deviation
Sox2: SRY (sex determining region Y)-box 2
TERT: Telomerase reverse transcriptase
TUNEL: Terminal deoxy nucleotidyl transferase-mediated nick end labeling
vWF: Von Willebrand factor
