# Supplementary figures and images for: Grafted c-kit+/SSEA1− eye-wall progenitor cells delay retinal degeneration in mice by regulating neural plasticity and forming new graft-to-host synapses
Source: Stem Cell Res Ther. 2016 Dec 30;7:191. doi: 10.1186/s13287-016-0451-8 (PMC5203726; doi:10.1186/s13287-016-0451-8)

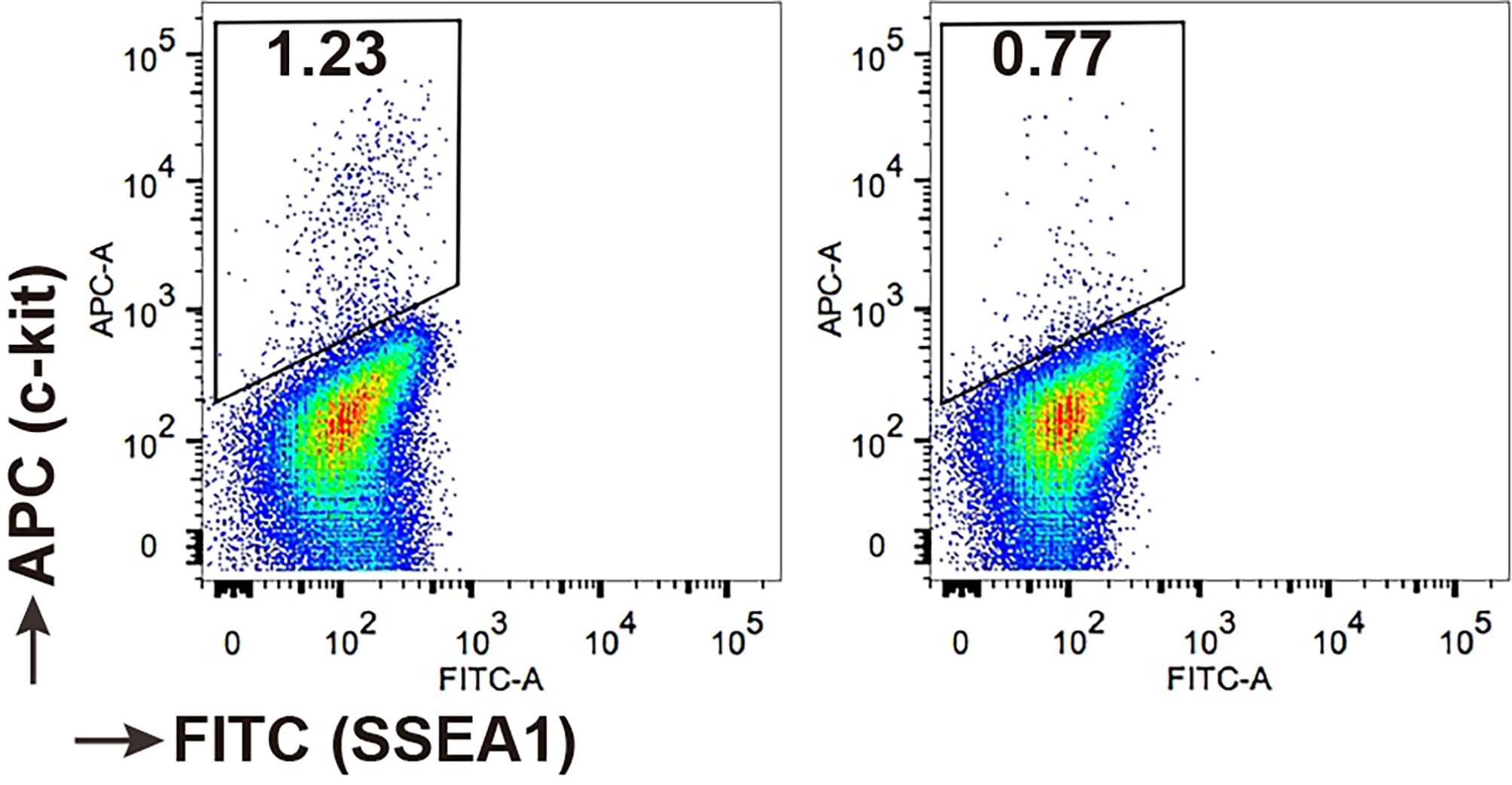

Supplement: Additional file 1: Figure S1. — Fluorescence-activated cell sorting of c-kit+/SSEA1− cells. C-kit+/SSEA1− cells were isolated in vitro illustrated by flow cytometry. (PNG 810 kb) [file 13287_2016_451_MOESM1_ESM.png]

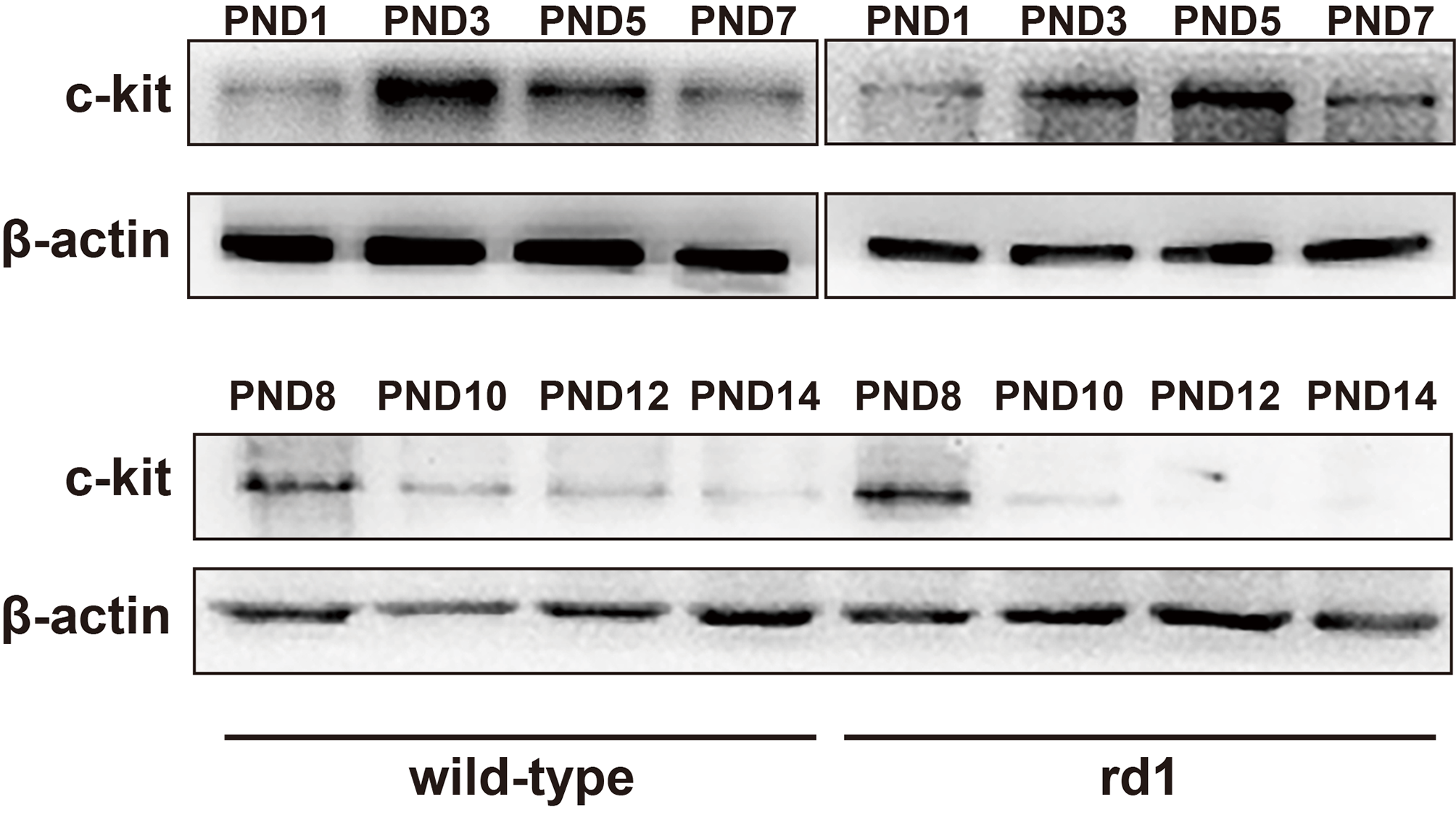

Supplement: Additional file 2: Figure S2. — C-kit expression in retina of wild-type mice and rd1 mice. Western blot analysis for c-kit expression in retinas at postnatal day (PND) 1, 3, 5, 7 (upper panel), 8, 10, 12, 14 (lower panel). (PNG 769 kb) [file 13287_2016_451_MOESM2_ESM.png]
